# Cerebellar Transcranial Direct Current Stimulation Modulates Anticipatory Postural Adjustments in Healthy Adults

**DOI:** 10.1007/s12311-023-01535-3

**Published:** 2023-02-23

**Authors:** Haian Mao, Wenwu Xiao, Zengming Hao, Shengjun Wen, Huaichun Yang, Fahad Sultan, Chuhuai Wang

**Affiliations:** 1 Department of Rehabilitation Medicine, The First Affiliated Hospital, Sun Yat-Sen University, Guangzhou, China; 2 Department of Cognitive Neurology, Hertie Institute for Clinical Brain Research, Otfried-Müller Str. 27, 72076 Tübingen, Germany; 3 Department of Integrative Medical Biology, Umeå University, Johan Bures Våg 12, 901 87 Umeå, Sweden

**Keywords:** Anticipatory postural adjustments, Cerebellum, Bilateral rapid shoulder flexion, Transcranial direct current stimulation

## Abstract

During forward swinging of the arm, the central nervous system must anticipate the effect of upraising upon the body. Little is known about the cerebellar network that coordinates these anticipatory postural adjustments (APAs). Stimulating different cerebellar regions with transcranial direct current stimulation (tDCS) and with different polarities modulated the APAs. We used surface electromyography (sEMG) to measure muscle activities in a bilateral rapid shoulder flexion task. The onset of APAs was altered after tDCS over the vermis, while the postural stability and the kinematics of arm raising were not affected. To our knowledge, this is the first human cerebellar-tDCS (c-tDCS) study to separate cerebellar involvement in core muscle APAs in bilateral rapid shoulder flexion. These data contribute to our understanding of the cerebellar network supporting APAs in healthy adults. Modulated APAs of the erector spinae by tDCS on the vermis may be related to altered cerebellar brain inhibition (CBI), suggesting the importance of the vermal-cerebral connections in APAs regulation.

## Introduction

Postural control is vital for body stabilization and equilibrium during everyday life [1]. Anticipatory postural adjustments (APAs), prior to self-initiated movements, and compensatory postural adjustments (CPAs) serve to maintain postural stability by anticipating and compensating for destabilizing forces associated with moving limbs [2, 3]. The programs in the cortical networks execute APAs and CPAs that are optimal for the achievement of goal-directed movements [4]. The cerebellum is reciprocally connected with the cerebral cortex [5, 6] and may affect these processes. Indeed, one fMRI study by Schmitz et al. reported that APAs were associated with the activation of the cerebellum, sensorimotor areas, and somatosensory area [7]. Thach previously observed early dentate discharge preceding motor cortex activity before monkeys made their desired movements [8]. The observation of intralimbic APA disruption in cerebellar ataxic patients strengthened evidence for a role of the cerebellum in APAs [9]. Thus, we hypothesize that the cerebellum participates in APAs and that cerebellar interference would interrupt the APAs.

The present study was designed to verify cerebellar involvement in APAs in healthy adults. For this purpose, we applied tDCS on the vermis or right cerebellar hemisphere to influence cerebellar activity. The participants were asked to perform a bilateral rapid shoulder flexion task standing on a force plate before, during, and after tDCS. The postural parameters and the muscle activity from the anterior deltoid (AD) and erector spinae (ES) were recorded simultaneously. The onset of burst activity in postural muscles (ES) with respect to AD activity, an index of anticipatory postural adjustment abilities, was computed offline and compared among the three sessions. Specifically, we wanted to address the following issues: first, whether cerebellar perturbations also perturb the APAs of core muscles; second, whether the APAs are altered differentially depending on the c-tDCS mode; and third, whether there are regional differences in the cerebellum regarding APA regulation.

We demonstrated the following characteristics of the muscle activation patterns in the bilateral rapid shoulder flexion task before, during, and immediately after c-tDCS in human participants. First, the relative activation onsets of both arms were not affected by the c-tDCS. Second, the activation of the ES preceded that of the AD with an average of 35 ms, and this parameter was shortened to 16 ms by cathodal c-tDCS on the posterior vermis, while anodal c-tDCS seemed to have the opposite effect. Anodal or cathodal c-tDCS on the right hemisphere had no detectable influence. Third, the postural parameters were not affected by c-tDCS or by altered APAs. These results suggest that the cerebellum, specifically the vermis, participates in anticipatory postural adjustments for voluntary movement. The shorter response time of the ES during APAs caused by vermal tDCS may be related to reduced cerebellar brain inhibition (CBI) that exerts excitatory control over the core muscles, suggesting the importance of vermal-cerebral connections in APA modulation [10, 11]. Our results are important for future studies on whether c-tDCS can help to improve postural ataxia in cerebellar patients.

## Materials and Methods

### Participant Enrollment

The sample population was recruited from the student and staff population of the hosting institute and from the community. Advertisements were displayed and circulated on social media platforms. Healthy adults from age 18 to 60 years were included. Participants were excluded if they met any of the exclusion criteria, including (1) a history of a neurological disorder, such as chronic pain or seizures, metallic implants in the brain/skull, a pacemaker in the heart, or regular medications and (2) pregnancy. In total, 50 healthy right-handed adults were recruited in this study. They were randomly and equally assigned to the (A) vermis anodal group, (B) vermis cathodal group, (C) right hemisphere anodal group, (D) right hemisphere cathodal group, or (E) sham group, among which includes 5 participants were sham-stimulated on the vermis and another 5 on the right cerebellar hemisphere.

### Postural Control Task

In the present study, the internal perturbation task of bilateral rapid shoulder flexion was adopted as the postural control task. During the task, participants stood on the force plate (ProKin 252, TecnoBody, Italy) with their feet shoulder-width apart and their arms relaxed and hanging downwards. Once the visual cue was given, the participant was asked to raise both arms forward as fast as possible to approximately 90 degrees followed by arm lowering to the starting point, getting ready for the next trial. The visual cue, “a red dot on a black background,” was displayed every 5 s and lasted for 1 s.

### Surface Electromyography (sEMG)

The muscle activities were recorded by a 16-channel surface electromyography (sEMG) system (Myomonitor IV, Delsys, USA) to obtain the APA and CPA capacity. Since both arms were raised during the task, electromyography (EMG) activity was recorded from the anterior deltoid (AD) of both sides as the prime mover muscles and the erector spinae (ES) at the 12th thoracic level of both sides as postural muscles, according to previous APA studies [12]. The sEMG sensor attachment sites for the ES and AD were identified 10 cm below the end of the lateral clavicle and 3 cm lateral to the 12th thoracic vertebra, respectively. Prior to sensor placement, the skin surface was treated with high-chloride abrasive electrolyte gel to lower skin impedance. Impedances were kept below 5 kΩ. The acquisition frequency used was 1214 Hz, as indicated on the packaging of the main amplifier (Myomonitor IV, Delsys, USA), with a gain set to 1000 times, bandpass frequency at 20–450 Hz, 16-bit resolution, and 1.2 μV of noise.

### Cerebellar Transcranial Direct Current Stimulation (c-tDCS)

To target the vermis or right cerebellar hemisphere with tDCS (model EM8060, E&M Medical Tech., China), the active electrode (square-shaped conductive rubber electrodes, 4.3 cm × 6 cm, embedded in saline-soaked sponges (5 cm × 7 cm)) was placed vertically with its lower boundary 2 cm below the inion along the middle line or 3 cm lateral right to the middle line, while the reference electrode was placed over the superior aspect of the right trapezius muscle [13, 14]. The stimulation was delivered at 2 mA for 20 min. The stimulation current was gradually ramped on and off over 30 s. In the control group, five participants received sham stimulation with one electrode over the vermis and the other five on the right cerebellum with the same electrode placements as the experiment groups. The current was ramped up for 30 s, then stayed at 1 mA for 10 s, and ramped down for another 30 s.

### Experimental Procedures

All participants received 20 min of c-tDCS (2 mA) or sham stimulation on the vermis or right hemisphere according to the group assignment. Each volunteer completed 3 sessions, 10 trials each, of the bilateral rapid shoulder flexion task before, during, and immediately after c-tDCS on the force platform. Throughout the experiment, participants were asked to make upward arm movements to their shoulder level as fast as they could and to maintain posture as balanced as possible. Prior to the start of the experiment, each participant performed 3 trials as practice.

### Data Analysis

**Fig. 1:**
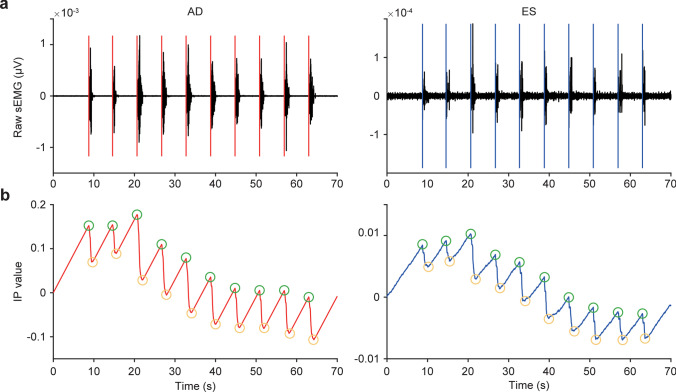
Example of the IP method to detect AD and ES muscle activation onsets in one participant. **a** Raw sEMG traces with AD muscle activation onset marked by red vs. ES muscle activation by blue vertical lines. **b** The corresponding IP values. The peaks are marked by green circles, while the bottoms are marked in orange. The X-axis indicates the time in seconds

The sEMG signal was filtered with a fourth-order Butterworth bandpass filter (60–500 Hz) and subsequently rectified. Onsets of muscle activation were determined with the integrated protocol (IP) and are based on work by Santello and McDonagh and Allison [15, 16]. Given an sEMG time series S(t), the onset of muscle activation was calculated by performing a continuous integration of the rectified samples (IP(t)). A linear function R(t) was used to create a reference line going to the same final value, IP(L), which is the maximum of IP(t). Consequently, the onset time of muscle activation could be determined as the time point, t0, at which R(t) and IP(t) yield the maximal difference (Fig. 1) [17].

$$\begin{array}{c}IP\left(t\right)=\sum_{i=0}^t\left|\mathrm S\left(\mathrm i\right)\right|,t=1,2,\dots,\mathrm L.\\\begin{array}{cc}R\left(t\right)=\frac{IP\left(L\right)\;\ast\;t}L,&t=1,2,\dots,L.\end{array}\end{array}$$

Time 0 (T0) was defined as the onset of activity of the left or right deltoid muscle, and the onset of activity of the left and right ES was expressed relative to T0. To calculate T0 in bilateral shoulder movements, we used both the left and right anterior deltoid muscles for shoulder flexion and for analysis. The mean onset time of the 10 trials for each muscle was calculated and used in the analysis. We defined the onset of activity of each muscle − 250 ms before T0 or within + 50 ms as feedforward activation [18] and after T0 + 50 ms to + 350 ms as feedback activation. Figure 1 shows typical raw sEMG data and IP values for the left AD and left ES [19, 20]. The APA and CPA values that fell outside these two ranges were excluded from the analysis.

### Statistical Analysis

Descriptive statistics are reported as the mean ± standard deviation (SD). Differences in age, height, weight, and body mass index (BMI) were analyzed using one-way ANOVA in MATLAB (R2022a, The MathWorks Inc., Natick, MA, 2000). Differences in baseline levels of APAs, CPAs, swing area, and swing length were compared between groups with independent-sample Kruskal‒Wallis *H* tests. APA and CPA changes between sessions within groups were compared with related-sample Friedman’s two-way analysis of variance by ranks. Kruskal‒Wallis and Friedman’s tests were performed using IBM SPSS (version 20.0, IBM Corp., Armonk, NY, USA). The results were considered significant at *p* < 0.05.

## Results

### Sample Populations

Fifty participants (25 females and 25 males) completed the experiment. The demographic characteristics of the sample populations are shown in Table 1.

**Table 1:** The demographic characteristics of the sample populations

Participants	Vermis anodal	Vermis cathodal	Hemi anodal	Hemi cathodal	Sham	*F*	*p*
Sex (f/m)	10 (5/5)	10 (5/5)	10 (5/5)	10 (5/5)	10 (5/5)	NA	NA
Age (years)	26.7 ± 5.98	30.9 ± 4.86	27.9 ± 5.07	27.70 ± 6.06	27.40 ± 3.81	0.96	0.44
Height (m)	1.69 ± 0.09	1.69 ± 0.11	1.66 ± 0.07	1.64 ± 0.07	1.69 ± 0.12	0.56	0.69
Weight (kg)	65.40 ± 13.27	64.10 ± 15.57	66.00 ± 17.06	58.40 ± 9.49	61.90 ± 11.68	0.51	0.73
BMI (kg/m^2^)	22.69 ± 2.66	22.29 ± 4.08	23.60 ± 4.56	21.65 ± 2.42	21.58 ± 1.66	0.65	0.63

Descriptive statistics are reported as the mean ± SD. The *F* and *P* values were obtained from one-way ANOVA

### Behavior Results

#### Bilateral Rapid Arm Flexion

**Fig. 2:**
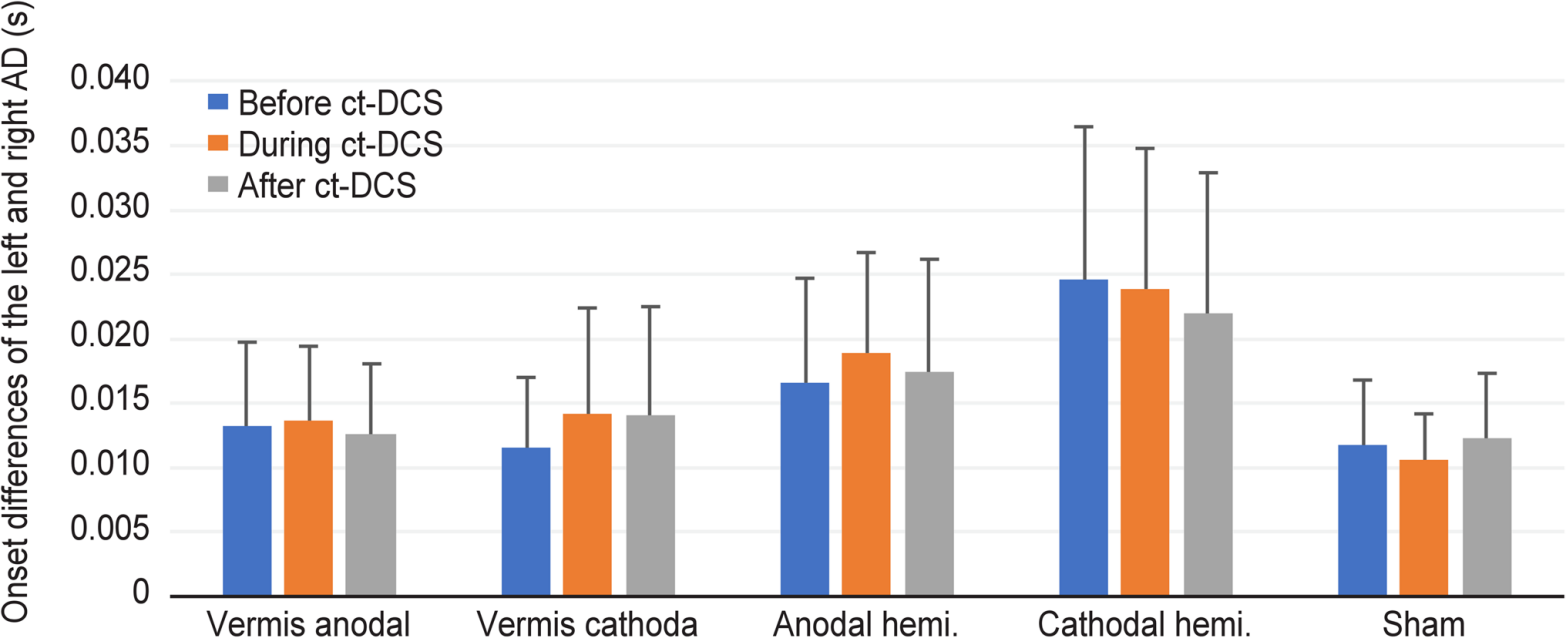
The time difference of movement onset of both arms. Note that the time lags of arm activation were in the range of 5 to 30 ms, and the average range was approximately 15 ms

All the participants completed the rapid bilateral arm flexion task without postural problems. The onset of activity of the left and right AD had a difference of 15 ms on average, without any obvious dominances (Fig. 2). Note that the gap was largest in the cathodal hemisphere group and smallest in the sham group. However, the pattern was not affected by c-tDCS in any group (Friedman’s test, *p* > 0.05).

#### Postural Balance During Bilateral Rapid Arm Flexion

**Fig. 3:**
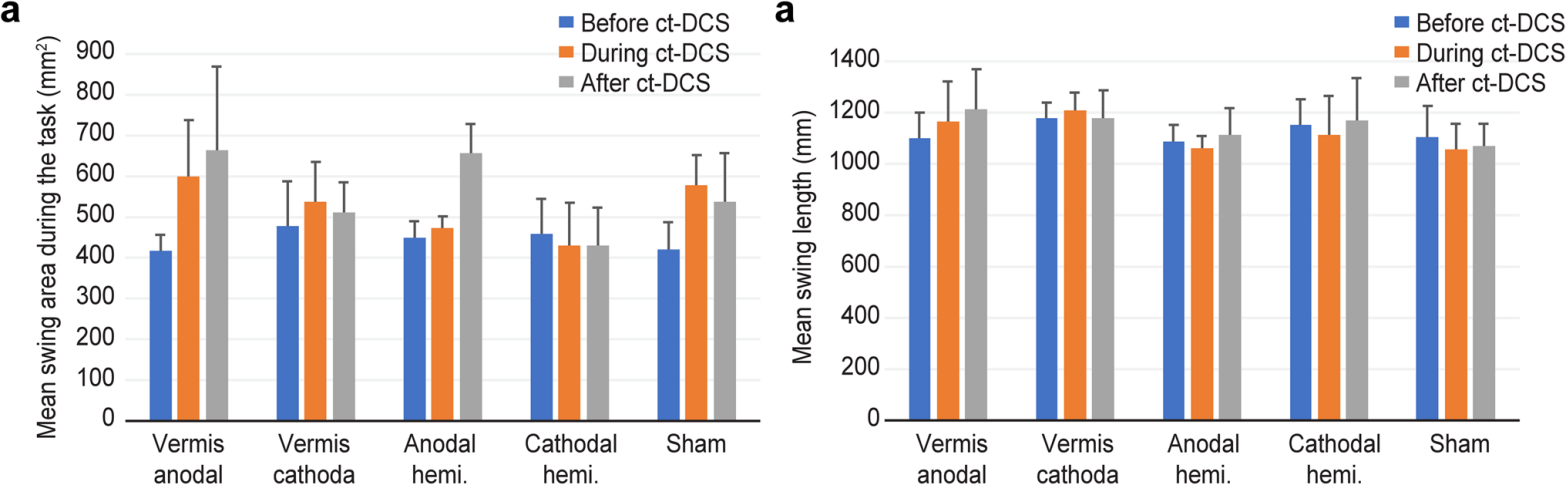
The postural parameters during the bilateral rapid arm flexion task before, during, and immediately after cerebellar tDCS or sham stimulation in different groups. **a** The swing area and **b** the swing length were not significantly different

Balance data were recorded from 32 participants. The baseline body swing area and swing length did not differ among groups before c-tDCS (Kruskal‒Wallis test, *H*: 0.223, df: 4, *p*: 0.994 and *H*: 1.792, df: 4, *p*: 0.774, respectively). There was no significant effect of session (Fig. 3, Friedman’s test, *p* > 0.05). A single session of c-tDCS had no effect on balance control during bilateral rapid arm flexion.

### Anticipatory and Compensatory Postural Adjustments (APAs and CPAs, Respectively)

**Fig. 4:**
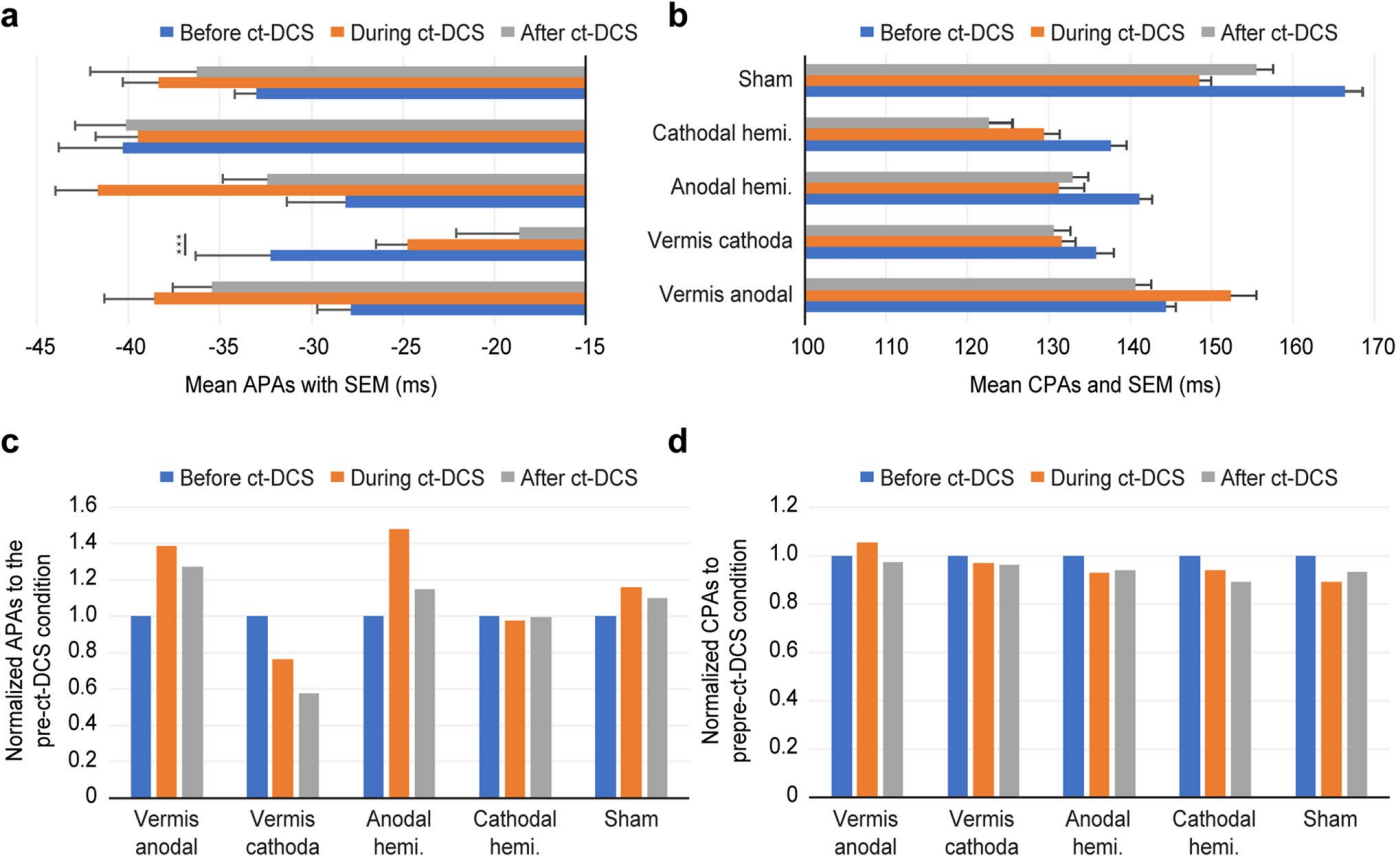
The difference in the influence of tDCS on APAs and CPAs between the ES and AD. **a** APAs were significantly shortened in the vermis cathodal group. **b** CPAs were not significantly affected by c-tDCS in any group. Note that the averaged APAs before tDCS within the vermis cathodal group were approximately − 32 ms and were shortened to − 18 ms after c-tDCS, while within the vermis anodal group, they were approximately − 27 ms and − 35 ms, respectively. **c** The normalization of APAs to the baseline APAs. **d** The normalization of CPAs to the baseline CPAs

The baseline APAs and CPAs differed among groups (Table 2). The findings of the Bonferroni post hoc analysis suggested that the significance came from comparisons of the sham group to the other groups. The respective Friedman test for multiple related samples revealed a significant effect of session in the cathodal group on the vermis and anodal group on the right hemisphere (Table 3). Specifically, the APAs between the right ES and the left AD as well as the APAs between the left ES and the right AD were significantly shortened after c-tDCS on the vermis (post hoc analysis Bonferroni, *p* = 0.001). The APAs from the right ES and left AD and those from the right ES and right AD in the anodal hemisphere group were significantly modulated during the c-tDCS but did not differ between the pre- and post-c-tDCS sessions (*p* = 0.092 and *p* = 0.086, respectively). The averaged APAs from all four pairs are summarized and compared in Fig. 4a. There was a large variability in CPAs among groups (Table 2). However, there was no significant effect of sessions in any group (Fig. 4b). When the APAs and CPAs were normalized to the baseline condition within each group, the vermis cathodal group showed a decreased value while the anodal groups showed opposite trends (Fig. 4c). However, there was much smaller variability in CPAs (Fig. 4d).

**Table 2:** Results of the Kruskal‒Wallis test for the comparison of baseline APAs and CPAs among groups

		Left ES–left AD	Right ES–left AD	Left ES–right AD	Right ES–right AD
APAs	*H* (df)	7.14 (4)	14.11 (4)	8.457 (4)	11.736 (4)
	p value	0.129	0.007	0.076	0.019
CPAs	*H* (df)	31.462 (4)	11.49 (4)	27.879 (4)	11.069 (4)
	p value	< 0.001	0.022	< 0.001	0.026

**Table 3:** Results of Friedman’s test for the comparison of APAs among sessions within groups

		Left ES–left AD APAs	Right ES–left AD APAs	Left ES–right AD APAs	Right ES–right AD APAs
Vermis anodal	*F*_*F*_ (df)	4.545 (2)	5.768 (2)	5.6 (2)	2.212 (2)
	p	0.103	0.056	0.061	0.331
Vermis cathodal	*F*_*F*_ (df)	3.225 (2)	11.221 (2)	12.761 (2)	2.257 (2)
	*p*	0.199	**0.004** **pre-post: 0.001**	**0.002** **pre-post: 0.001**	0.323
Hemi. anodal	*F*_*F*_ (df)	0.966 (2)	9.0 (2)	2.552 (2)	13.5 (2)
	*p*	0.617	**0.011** pre-post: 0.092	0.279	**0.001** pre-post: 0.086
Hemi. cathodal	*F*_*F*_ (df)	1.443 (2)	3.505 (2)	1.287 (2)	1.60 (2)
	*p*	0.486	0.173	0.527	0.449
Sham	*F*_*F*_* (df)*	1.161 (2)	3.121 (2)	1.623 (2)	4.508 (2)
	*p*	0.56	0.21	0.444	0.105

A nonparametric Friedman test was conducted to measure the level of APAs. Pairwise multiple comparisons with a Dunn–Bonferroni post hoc test were performed if Friedman’s test indicated significance. Significance (*p* < 0.05) is indicated in bold

## Discussion

The current study represents, to our knowledge, the first evidence from healthy adults pertaining to cerebellar modulation of APAs. By targeting specific regions over the cerebellum using tDCS in a bilateral rapid shoulder flexion task, we were able to identify that the vermis is specifically involved in APAs. In addition, tDCS on the vermis area was able to modulate the APAs in this task, with cathodal tDCS significantly impairing APAs while anodal tDCS enhanced the motor response. There was, however, no such effect for tDCS over the right hemisphere. Furthermore, postural stability was not significantly affected by cerebellar tDCS. In the following, we will first compare our findings to previous results from tDCS studies on APAs before we return to discuss the implications of our findings on the brain network of APAs.

In our study, we found that tDCS over the vermis modulates APAs in a rapid bilateral shoulder flexion task, while tDCS targeting the right cerebellar hemisphere had no obvious effect. This finding is in agreement with the observation that cTBS over lobe VIII did not significantly modulate the amplitude and timing of APAs during gait initiation [21]. Furthermore, due to methodological limitations, MEG may also miss cerebellar activity during the APA phase [22]. However, the cerebellum can be reliably identified by means of anatomical navigation; its activity can be selectively modulated by noninvasive brain stimulation, such as tDCS and TMS; and the effects of cerebellar stimulation can be reliably assessed by EEG [13, 23–27]. The effect of c-tDCS on the motor cortex was different depending on the polarity, in which cathodal tDCS resulted in a decrease in excitability while anodal tDCS increased excitability [28, 29]. In chronic nonspecific LBP patients, it was observed that the increased excitability of the motor cortex coincided with impaired APAs [30, 31]. In our study, cathodal c-tDCS might have increased motor cortex excitability and impaired APAs in healthy adults. However, anodal c-tDCS had no significant effect on APAs, arguably due to anatomical differences between the cerebellum and cerebral cortex, and the anode of tDCS is not effective in changing the direction of behavior in healthy volunteers [32, 33].

On the other hand, there were no significant effects of c-tDCS on center of gravity sway parameters during the bilateral rapid arm flexion task in our study. It was reported that anodal tDCS to the cerebellum promoted motor learning and gait adaptation [34, 35]. In addition, cerebellar activity increases during postural adjustments [36, 37]. In this study, however, on the one hand, all the participants were healthy men, and the degree of difficulty of the task was low (standing with eyes open and legs closed). The absence of any need to learn or adapt may have contributed to the lack of effect of cerebellar tDCS on postural control and balance. On the other hand, the participants were told to remain as balanced as possible to elicit APAs. As a result, in our setting, c-tDCS did not affect the standing posture control of healthy participants.

One of the limitations of this study is that all participants were healthy young adults. The main finding of this study is that ES APAs changed after c-tDCS over the vermis, while postural control remained. However, it is unclear whether c-tDCS could rescue the impaired APAs in chronic nonspecific LBP or cerebellar ataxia patients. Further studies are needed to apply the c-tDCS experimental procedures to those with abnormalities in APAs.

It is also unclear whether increases or decreases in APA latencies have beneficial or deleterious effects on standing posture control. Although both total swing length and swing area were not significantly affected after c-tDCS, it is unknown how APAs and postural control were differentially modulated in the cerebellum. Thus, it is necessary to assess postural parameters in addition to task-related periods in future studies.

## Conclusion

Our findings obtained with healthy adults showed significant APA changes after cathodal c-tDCS over the vermis. Of particular interest is that the anodal c-tDCS over the vermis has an opposite but not significant effect on the APAs. We propose that the vermis is the cerebellar area that is involved in the regulation of APAs. Further studies are needed to look for effective stimulating methods to the cerebellum and investigate the subsequent changes in the cerebral cortex. Such studies will enhance our understanding of cerebellar mechanisms of postural control in healthy humans and provide interventions for postural dysfunction, such as chronic low back pain and cerebellar ataxia patients.

## Data Availability

The data and materials in the paper are available upon request.
